# The Hospital. Nursing Section

**Published:** 1906-12-08

**Authors:** 


### The Hospital.

### Nursing Section.

Contributions for "The Hospital," should be addressed to the Editor, "The Hospital" Nursing Section, 28 & 29 Southampton Street, Strand, London, W.C.

No. 1,055.—Vol. XLI. SATURDAY, DECEMBER 8, 1906.

### Notes on News from the Nursing World.

### LADY MINTO AND HER NURSES.

We understand that the first four nurses have been selected to work under the Indian Nursing Association as organised by the Countess of Minto, and will start for India on the 28th inst. It is a guarantee of their capacity and character that they were chosen after much careful consideration by Miss Sidney Browne, R.R.C., late Matron-in-Chief of Queen Alexandra's Imperial Military Nursing Service, assisted by Mrs. Shepherd, whose name is so well known in connection with the Up-country Nursing Association, now merged in the new organisation.

### QUEEN'S NURSES AND THE OUTDOOR POOR.

The "Nursing of the Outdoor Poor" was one of the subjects discussed on Friday last at the Poor-law Conference for the South-Eastern and Metropolitan Poor-law Division. It was introduced by Miss Amy Hughes, who, having described the development of district nursing, proceeded to refer to the Local Government Board Order of 1892, under which Boards of Guardians may appoint district nurses and make regulations for the performance of their duties according to the directions of district medical officers. Miss Hughes said that advantage had been taken of the Order in a number of Unions; but that as no special training and no expert nursing supervision were given, the results had not been altogether satisfactory. She referred to the fact that in Lancashire and Yorkshire, after the plan had been fairly tried, it had been discontinued in favour of contributing to the local associations employing Queen's Nurses, and she urged that grants from the Guardians to the Institute and its branches would complete a scheme to bring skilled nursing to the poorest people. As our columns show from time to time, the policy advocated by Miss Hughes is steadily gaining ground, and there is no doubt that, both in the interests of the patients and of nurses, it is preferable to the policy which was initiated by the Local Government Board Order of 1892, and which failed, because the conditions necessary to success were not observed.

### VICEREGAL INTEREST IN NURSING.

Lord and Lady Aberdeen are continuing the good work done by Lord and Lady Dudley in Ireland by showing practical interest in nursing movements. We mentioned last week that Lady Aberdeen attended the opening meeting in Dublin of the winter session of the Irish Nurses' Association, which seems to be steadily growing in numbers and in influence. In the North of Ireland the Viceroy and his wife recently visited the Jubilee Nurses' Home at Armagh, where they were received by the President, Miss Alexander. Lord Aberdeen delivered an encouraging and sympathetic little speech, in the course of which he spoke of the benefits derived by poor people from the ministrations of the nurses, and assured Miss Alexander and the Committee that Lady Aberdeen and himself greatly appreciated the cordial welcome given them.

### MARRIAGE A DISQUALIFICATION.

At the meeting of the London County Council last week, Sir Melville Beachcroft called attention to the fact that the Education Committee proposed that the person appointed to the post of superintendent nurse in the education branch of the Public Health Department should be required to resign in the event of marriage. He said that he had yet to learn that marriage was a disqualification for the service of the Council, and moved that the proposal should be referred back. Mr. J. Whittaker Thompson and Sir Thomas Brooke-Hitching supported the motion, the latter observing that the officer would be all the better able to cope with her duties if she were married. We do not go as far as this, but we regret that a majority of the Council approved the condition laid down by the Education Committee. Marriage should neither be a qualification nor a disability for the post in question. The vital point is the adequate performance of the duties of superintendent of nurses, and the selection of a highly trained woman of ripe experience.

### INSULTS TO NURSES.

A report is given in the *Skibbereen Eagle* for November 24 of some extraordinary proceedings which took place at a meeting of the Bandon Guardians. The occasion was the appointment of a fully trained nurse, and the three candidates for the post were in attendance. One of these was described by the clerk as "a very nice-looking girl," the remark being received by the Guardians with laughter instead of by a reprimand. This was before the candidates were called in, but on their admission to the room the clerk observed, "They are rather smart and good-looking"; whereupon the Guardians are reported to have indulged in laughter for the second time. In the end the only one of the three candidates who had not completed her period of training was elected, the proposer observing that she was "the daughter of a large ratepayer in the Union." This is a matter for the Irish Local Government Board, whose sanction to the appointment is essential, to consider. We protest, however, both against the comments in which the clerk indulged and against the unseemly demonstrations with which they were greeted. What would these men have said if their daughters or sisters had been treated in the same manner?

### THE SUICIDE AT AN ISOLATION HOSPITAL.

The inquiry into the death of a nurse at Havant Isolation Hospital who had poisoned herself with morphia calls for remark. It was clearly established that she had no ground of complaint as to the manner in which she had been treated by the authorities or by her fellow-nurses; and she left a letter behind her requesting that some of her belongings should be given to the latter for their kindness to her. But how did she get possession of the tubes containing the morphia by means of which she put an end to her life? As the acting medical officer stated at the inquest, a nurse ought never to be allowed to carry any poisonous drugs about with her. Moreover, the law requires that the name of the vendor shall always be attached to poisons, and in this case it was not. Did no one at the Havant Isolation Hospital know that Nurse Lawson had such things in her possession, nor where she obtained them?

### THE DESIGNATION OF NURSE.

It is an unusual experience for a trained nurse attending a private case to find that the patient has an insurmountable objection to her ordinary designation. We hear, however, of such an instance from a correspondent whose patient insists upon calling her by her surname. When alluded to in the house she is spoken of not as "Nurse," but as "Miss ——." The reason given by the patient is that in the past she has been "sickened of nurses." In such a simple matter as this a patient may assuredly do as she pleases, but as the lady in this case confesses that she gets on very well with her present nurse, her prejudice will probably soon be dissipated.

### ATTEMPT TO IMPOSE A RELIGIOUS TEST.

The religious question was raised at meetings of the Liverpool Select Vestry and the York Guardians. At Liverpool there appears to have been a misunderstanding. It occurred in reference to the election of night superintendent at the Workhouse Hospital, an allegation being made that Miss Stewart, the superintendent of nurses, had discouraged the application of a second Protestant candidate on the ground that the nomination would "split the Protestant vote." Asked for an explanation, Miss Stewart simply declared the allegation to be untrue, and the candidate recommended by the Workhouse Committee was appointed. At York, however, one of the Guardians actually moved that the Board should advertise for a Protestant superintendent nurse because friction has taken place among the chief officials of the workhouse owing to religious differences. We are glad to say that the motion was defeated by eighteen votes to two, the great majority endorsing the view that the institution being a public one, anyone has the right to apply for the position of superintendent nurse. That an attempt should seriously be made to impose a religious test is amazing.

### NURSES AND POLITICS.

A very unusual reason was given at the annual meeting of the Warrington Nursing Association for the decrease in the amount of subscriptions. The chairman, in moving the adoption of the report, stated that during the past general election there was a rumour that the then official nurses of the Association interfered in politics; and in consequence several subscriptions had been withdrawn. He described the rumour as "tittle tattle," and we conclude that it was absolutely unfounded. Also that it had nothing to do with the resignation of Miss Whitehead, the superintendent, for which the chairman said the committee knew of no valid reason. The incident, however, strongly proves, if proof were needed, the importance of nurses engaged in district work refraining from identifying themselves with party politics. They are, of course, entitled to their own convictions on every question, but they cannot be too careful not to commit themselves to the advocacy of any political cause. The nature of their work is in itself a prohibition.

### THE TRAINING AT BRIDGWATER UNION HOSPITAL.

A great compliment has been paid to the authorities of Bridgwater Union Hospital by Dr. Fuller, Local Government Medical Officer, and Mr. Preston Thomas, Local Government Board Inspector. At the last meeting of the Bridgwater Guardians Mr. Preston Thomas observed that in Dr. Fuller's report of that institution he stated that there was nothing at all to find fault with, and that it was a treat to go over a workhouse where everything was so well managed as it was there. Dr. Fuller thought that the training of the probationers was most satisfactory, though it required a good deal of trouble both on the part of the doctor and of the superintendent nurse. He added that the system which was being carried out in that house with so much advantage was working out exceedingly well, and was for the benefit of everyone concerned. Mr. Preston Thomas congratulated the Guardians upon these statements, which, from his own experience, he entirely endorsed. The training school at Bridgwater was established in 1904, and its recognised condition of efficiency is largely due to the appointment of Miss M. Barnes as superintendent nurse in May of this year.

### AN INQUIRY AT KING'S LYNN.

By a majority of one, the King's Lynn Guardians decided at their last meeting to delegate to a committee the task of an inquiry as to the efficiency of the nurses in the Workhouse Infirmary with regard to number or condition of the inmates. That this investigation is necessary was shown by the Rev. A. H. Hayes, who, returning to the charge, affirmed that the recent reduction of the staff had not contributed to either efficiency or economy. Mr. Hayes pointed out that, while the salary of the nurse who had been dispensed with was £28 a year, the

Guardians had expended since August on extra nursing assistance £11, as well as £8 8s. for a special nurse for a typhoid case, and that they now proposed the engagement of a temporary nurse at a salary of £1 11s. 6d. per week. As Captain Hervey, the Local Government Board inspector, has already affirmed in reference to King's Lynn Infirmary, it is probably not more nurses, but more trained nurses who are wanted.

### IMPERIAL MILITARY NURSING SERVICE.

The following staff nurses have been promoted to the rank of sister in Queen Alexandra's Imperial Military Nursing Service: Miss E. Barber, Miss A. E. FitzGerald, Miss E. C. Fox, Miss A. M. MacCormac, Miss M. O'C. McCreery, Miss F. M. MacGregor, Miss M. MacGregor, Miss M. E. Neville, and Miss E. M. Robinson. We are also officially informed that Miss E. M. M. Malim, Miss M. E. Smith, Miss N. Stewart, and Miss S. W. Wooler have been appointed staff nurses.

### HOSPITAL NURSES AND INVALID COOKERY.

The trays of invalid cookery eventually shown at the Cookery and Food Exhibition last week were extremely attractive in appearance. Tastefully decorated with vases of flowers and trails of smilax, they excited general attention. The trays were required to contain not less than four dishes of cooked food, one dish of fish, one light pudding, one jelly or custard and two beverages, including beef-tea. It was interesting to notice the ingenuity with which, in spite of these limitations, such a large variety of dishes were prepared. In Class 3—a special class open only to certificated nurses—Miss Cash, of Chelmsford Infirmary, carried off the gold medal. Her menu consisted of sole à la Hollandaise, caramel pudding, beef-tea, and wine jelly; and a most dainty and inviting collation it was. The nurses of Charing Cross Hospital must be congratulated on their success, for all the four exhibitors obtained an award. Their names are: Nurse Griffiths, silver medal; Nurse Mahony and Nurse Rivelt, bronze medals; and Nurse M. Green, certificate of merit and cookery book. The London Hospital nurses secured seven awards out of ten exhibits: Nurse Matthews, Nurse Malleson, and Nurse Michael, silver medals; Nurse Cater, Nurse Benson, and Nurse Scott, bronze medals; and Nurse Hope Bell, certificate of merit and cookery book. Nurse Walton, of Guy's Hospital, was awarded a silver medal; and Nurse Bodenham, also of Guy's, a certificate of merit and cookery book.

### POUND DAY IN A HAMPSTEAD HOSPITAL.

We are glad to hear that the Pound Day, which was organised by Miss Forster, matron of the hospital and Home for Incurable Children at Hampstead, and took place on Saturday, was very successful, especially seeing that it was a first attempt. During the day about 120 visitors called, and 451 lb. of goods, in value £8 5s. 6d., were received. In cash the sum of £11 16s. 3d. was realised after the payment of expenses for printing and advertising, but £2 of this amount is intended as the first contribution of an annual subscription. The matron hopes that the experiment will be the means of obtaining new friends and fresh interest for the institution, and she proposes to repeat it yearly. We have no doubt that next year Pound Day will bring in still more grist to the mill.

### MORE BEQUESTS TO NURSES.

The work of a nurse is often of a very disagreeable character, and, though faithfully performed, if often fails to receive even gracious recognition. It is therefore pleasing to notice that in two wills which have been proved during the past week it has been substantially acknowledged. In one case the late Mr. Henry Norris, who has left handsome bequests to several hospitals, has bequeathed the sum of £3,000 to Miss Emily Howard, formerly of the Westminster Hospital, "for her kindness and attention to him.". The late Sir Sydney Waterlow has left £200 to Miss Mansie Dales, of the Nursing Home, Hardwick Road, Eastbourne.

### MEDICAL MISSIONARY UNION.

The annual meeting of the St. Mary's Hospital Nurses' Branch of the Medical Missionary Union was held in the Board-room of the Hospital on Thursday evening last week, Captain Webbe in the chair. The report was read by the chaplain. There were fifty nurses present. Miss Bealey, late of Madras, spoke on the urgent need of English nurses to train native women.

### OUR CLOTHING DISTRIBUTION.

Although the winter continues mild on the whole, we hope that none of our readers will imagine that therefore the need for articles of clothing for patients in hospitals is not pressing. Between this and Christmas there may be a snap of very cold weather, and in any case we are not likely to get through January without frosts and snow, which will be severely felt by people who are not adequately protected against them. If we are to make a brave show on Tuesday, December 18, when the articles sent will be on view at the offices of The Hospital, between 3 and 5 p.m., we should still receive a substantial number of parcels. The matron of an East London hospital writes: "This year I see by your valuable paper that you have not received quite so many gifts as usual, but if it is in your power to grant me a small portion I shall indeed be grateful. For the past two years you have very kindly sent me a parcel, the contents of which gave our poor patients the greatest delight and pleasure. This is a very poor district, and we have several men and women from the ranks of the unemployed, who are very ill, and they do need 'something warm' to comfort them this Christmas." We have to acknowledge since last week contributions from Miss Taylor, Dulwich; Nurse L. L., Acton; Miss C. M. Jones, Woking; A. P., Newmarket; and Policy 311 National Pension Fund. The latest day for forwarding parcels is Monday, December 17, and they should be addressed to the Editor, 28 and 29 Southampton Street, Strand, London, with "Clothing Distribution" written outside.

### The Nursing Outlook.

"From magnanimity, all fears above;
From nobler recompense, above applause,
Which owes to man's short outlook all its charm."

### THE ASYLUM NURSE.

The rule we recommended many years ago in regard to attendants on the insane is now generally enforced. At that time it was the custom to distinguish the attendants on the men as male attendants, and those on the women as female attendants. With a view to the introduction of a careful system of training for attendants on the insane we recommended that they should be known as "attendants" and "nurses." By the asylum nurse is meant therefore a woman, not a man attendant. Looking back we are grateful to recognise the great improvements which have taken place both in the character and competency of those placed in charge of mental cases in asylums, especially in this country. We have always held the view that if everybody, both those who work in and are responsible for asylum administration, members of the medical profession and the public generally, could be brought to regard all mental cases as cases of illness, the general condition of the insane members of the community would be materially improved, and the chances of greater benefit from asylum, or, as we prefer to call it, hospital care must follow. This view is accepted by many of the leading alienists, and Dr. Clouston gave it practical expression at Morningside, where he placed all his probationers in the sick or infirm wards. Dr. Clouston's object was educational, and he succeeded in imbuing all who commenced their career, at Morningside, as attendants or nurses, with the idea, that, the patients who have to be cared for in asylums are ill with a bodily disease. No one with knowledge can underestimate the importance of getting this idea firmly fixed in each probationer's mind before he or she has had time to adopt the more common notion of being the keeper of a madman. The Medico-Psychological Association has done much useful service by instituting a system of examination for mental attendants and nurses and granting certificates. Some 8,000 such certificates have been issued, and about 1,000 probationers pass these examinations and are added to the number of certificated mental nurses annually.

It may be interesting to inquire how far the standard of mental nursing has been raised, to what extent the applicants for training are adequate to fill the vacancies which occur, and the quality of the material which medical superintendents have to shape into attendants and nurses. There can be no doubt that the most competent medical superintendents have secured the highest standard in the asylums under their control, and that the tendency is in this direction almost everywhere. On the candidates and their general qualifications an asylum chaplain has sent an interesting letter to *The Guardian*. As the result of thirteen years' observation this chaplain attaches the first importance to each candidate possessing a physique which will readily bear the strain of nursing the insane. He maintains that "experience has demonstrated that no class in society has a monopoly as regards physical fitness, which is of prime importance in choosing an asylum nurse. Candidates enter on probation, class distinctions are disregarded, and all are medically examined as to their general health. The intelligence of the attendant or nurse is regarded as an important factor in the attainment of efficiency in duty. Tact, sympathy, power of initiative in an emergency, are regarded as necessary qualifications for efficiency in this office." From this it would appear that difficulties are still experienced in finding a sufficient number of suitable candidates, for otherwise it would not be necessary to make such a point of physical fitness, which renders it essential, if vacancies are to be filled, that "most classes in society" shall be drawn upon. We may take it, therefore, that in practice this branch of nursing offers to the courageous and intelligent certain advantages, including a pension at the end of their service, which are not so readily obtainable in other portions of the nursing field.

The public, as represented by those who take an intelligent interest in public institutions, including the friends of the patients, are of course most interested in ascertaining whether the asylum nurse is efficient or not. We should say, as the result of our inspection of asylums and knowledge of the administration of these hospitals for the insane, that the asylum nurse is more efficient to-day, than ever before in the history of these establishments. The system of training pursued at most asylums, and the care which is exercised in the selection of the probationers, must militate in favour of a higher standard and greater efficiency. The system of training aims at emphasising and bringing into prominence the higher qualities of an efficient nurse. Training, too, obviates the old evils which permitted the novice to be left in sole charge of mental patients, for no charge nurse is now appointed, except she has proved herself to be experienced and capable. The system of supervision exercised over the whole staff is another guarantee that the average efficiency is high. By these means the status of the asylum nurse has been raised and is constantly tending to improve. We feel therefore there is good reason to believe in the efficiency of the average modern asylum nurse.

### The Care and Nursing of the Insane.

By Percy J. Baily, M.B., C.M.Edin., Medical Superintendent of Hanwell Asylum.

### II.—NURSING THE SICK.

(*Continued from page* 113.)

### 4. *The Patient's Toilet.*

Throughout the illness the patient's body must be kept scrupulously clean. In all cases the face and hands should be washed at least twice daily and the feet at least once daily. If the patient's strength will allow, he should be bathed at least once a week. In most cases of acute illness, however, this is neither allowable or possible, but in these cases the patient is to be washed all over while in bed every day, or at least twice a week. When the patient is very weak no attempt should be made to wash him all over at one time, but his body may be dealt with in sections, one part being done one day and another part another day. When a patient is about to be washed in bed his body linen is first to be removed and the bed is to be protected with a warmed blanket folded double, which should be passed beneath him. If the strength of the patient does not allow even this to be done, soft bath towels which have been previously warmed should be tucked in all around the part which is to be washed between the patient's body and the bed, the object of the towel being to soak up any water that may trickle down. This, however, should be prevented by the nurse wringing the flannel or sponge she is using as dry as possible. Each part of the patient is then to be washed with soap and water, and afterwards dried thoroughly before the next part is commenced. The drying should be effected with soft towels which have been previously warmed, or when the skin is very tender and red from pressure or threatening to break down into bed-sores, it will be better to use lint rather than towels. As soon as the washing in the bed is completed the patient should be covered with a warm blanket and allowed to rest for a time, after which his body linen should be again put on. Whenever a patient who is too weak to be taken out of bed and bathed is washed while in bed, two basins of water and two flannels should always be used. One basin is for use with the soap, and when as much of this as possible has been removed the part is then to be rinsed over with the clean water and the non-soapy flannel. The temperature of the water should be from 105° to 108°. The greatest possible care is to be exercised in thoroughly washing off the soap and in the subsequent drying.

In dealing with chronic and bedridden cases, especially in various forms of paralysis and in every case where the habits are faulty the patient should be put into a bath every day. Chronic bedridden cases require, if possible, even more care in the subsequent drying than do those suffering from acute illness on account of the greater tendency in these to the formation of bed-sores. The drying should never be hurried, and there should be no lack of clean, thoroughly dry, and warmed towels. The number of towels required for each patient will depend upon their size, but never less than two should be used. When the drying is complete, those parts of the patient's body which are specially subjected to pressure, such as the buttocks, sacrum, hips, etc., where bed-sores are likely to form, may be dubbed with lint soaked in methylated spirit. This strengthens the skin and ensures more thorough drying. The whole of the patient's body is then to be dusted over with some dusting powder, such as fuller's earth, a mixture of zinc oxide and starch, or powdered starch alone.

During the bathing careful note should be taken of the condition of the skin, so that the presence of any bruises, abrasions, or rashes may not be overlooked, but may be treated so as to prevent the development of bed-sores.

All parts of the body where two surfaces of skin come into contact with each other require the special attention of the nurse. In the armpits, the fold of the groin, between the buttocks and upper parts of the thighs, and in women beneath the breasts, especially if the patient happen to be at all fat, the secretions of the skin (sweat and sebaceous matter) are apt to be retained, when they are liable to become decomposed and set up irritation of the skin (eczema). The same thing is very liable to occur in paralysed patients whose limbs become contracted and crossed, and remain long in contact with one another leading to the formation of troublesome pressure sores. In these parts the skin requires to be treated, after washing and thorough drying, with some form of antiseptic dusting powder of which for this purpose boracic acid is undoubtedly the best.

The eyelids and mouth also require constant care from the nurse. In old people and those much exhausted by disease, the secretions from the eyes are apt to form crusts around the lids, and to stick them together. These should be removed with lint soaked in warm boracic lotion. If strong enough, the patient should use a tooth brush at least twice a day, but when he is unable to do this the teeth and lips must be cleaned by the nurse with a piece of lint moistened with warm water or warm boracic lotion. When the patient's temperature is high, when there is refusal or inability to take proper food, and in all cases of exhausting disease, the secretions of the mouth are very apt to dry and collect on the teeth and lips, forming brown and bad-smelling crusts or sordes, as they are called. In such conditions the tongue and lips are very likely to become cracked and painful. The accumulation of sordes should be prevented by washing the mouth with a piece of lint soaked in boracic acid lotion, as already recommended, and it may be grateful to the patient to squeeze a few drops of lemon juice into the solution. When the tongue and lips become cracked they should be painted with glycerin and borax by means of a camel-hair pencil.

The hair of female patients should be carefully brushed and then loosely plaited in two plaits. The first four or five inches of the hair nearest the scalp should remain unplaited, otherwise the thick plait between the patient's head and the pillow will prove very uncomfortable. In chronic cases, or in patients convalescing from acute conditions, when it becomes necessary to wash the head of a woman the hair must be dried as much as possible, and afterwards, when she is put back to bed, it should be combed over the pillow, which must previously have been covered with a dry warm towel. When it is quite dry it should be plaited. When there happen to be lice (pediculi) and nits in the hair, these must be got rid of at once. The lice are comparatively easily killed, but the removal of the nits is a much more difficult and tedious process. Only when the hair is in an utterly and hopelessly dirty state should it be cut off. The most simple method of killing the lice is to bathe the hair in a solution of carbolic acid (1 to 40). The hair must be thoroughly soaked in this solution. The best way to accomplish this is to draw the hair portion by portion through a sponge or flannel soaked in the lotion. It should then be tied up in some sort of cap: an ordinary triangular bandage does excellently for this purpose. On the following morning the hair should be washed with soap and water and afterwards combed through a small-tooth comb. Lysoform lotion (1 to 40), staphisagria ointment, white precipitate ointment, and ordinary paraffin lamp oil are also all of them excellent lice killers. They are to be used in precisely the same way as has been recommended for carbolic lotion, and the hair is afterwards to be treated similarly. White precipitate ointment is a powerful poison, and paraffin oil is highly dangerous on account of its inflammability; in lunatic asylums, therefore, it is wise not to adopt either of these two methods of treatment.

Nits, which are the eggs of the lice, are cemented to the hair, and are very difficult to dislodge. Their vitality should be destroyed by one or two applications of either of the methods suggested for killing the lice. They are said to be loosened from the hair by spirit of wine, but if this is so they still adhere sufficiently to make it impossible to remove them either by brushing or combing. Probably the best agent for loosening the nits is strong (glacial) acetic acid. The hair should be pulled through a small piece of lint or flannel moistened with this agent, which, if carefully used, does not appear to materially injure it. The only sure way, however, of removing the nits is to pick them off the hair individually, sliding them down the hair between the finger and thumb.

The nails of both hands and feet require to be kept clean and cut. This should be done after the weekly bath.

In dealing with patients who have received any kind of injury the greatest care must be taken over the removal of the clothing. The injured limb must be well supported during the process and frequently it becomes necessary to rip up the seams of sleeves or trousers. In any case it is a cardinal rule to remove the clothes from the uninjured side first.

*(To be continued.)*

Note.—Part I. of these lectures is published in book form, and can be obtained from the Scientific Press, Ltd., 28 and 29 Southampton Street, Strand, London, W.C. Price, 1s. net.

### The Nurses' Clinic.

### BLACKWATER FEVER.

Blackwater, or, to give it its full name, hæmoglobinuric, fever is the most dreaded of all malarial infections. It prevails chiefly in the malarial districts of Africa—*i.e.* in the Congo, the West Coast, and other parts—and is also met with in other tropical countries. It may occur in a susceptible subject during his first attack of fever, but as a rule the patient has already suffered from malaria several times before the especial symptoms of this disease make their appearance.

The attack begins with a rigor more or less severe, accompanied by vomiting and followed by fever, the temperature rising to about 103°.

The next morning the temperature will have fallen, probably after profuse perspiration, to 100°, or even to normal, but towards evening another severe rigor occurs, the vomiting returns, and the temperature rises higher than the night before.

After a day or two in this condition, or sometimes sooner, the patient experiences pain in the back and loins. At first it may be mere aching, but it soon becomes severe, and on passing water the urine is found to be dark brown or even black in colour.

The pain is felt either during a rigor or soon after the temperature has risen, and at the same time the patient will become very pale, the skin assuming a distinctly greenish yellow hue; the face looks pinched and sunken, the expression anxious.

Vomiting is frequent, and often accompanied by great pain; in severe cases it may become uncontrollable, and the patient dies from exhaustion.

In favourable cases the hæmoglobinuria lasts for two or three days, or even only 24 hours, at the end of which time the temperature falls and the symptoms begin to subside, the urine gradually returns to its normal colour, and the patient recovers.

The first thing for the nurse to do is to get the patient to bed between blankets (it is best to have a long mackintosh over the mattress, on account of the profuse sweating), with plenty of hot bottles. Hot drinks must be given if possible, but the patient may be too sick to allow of this at first. When the rigor is over and the hot stage reached, the hot bottles must be removed and also some of the extra blankets, but great care must be taken to avoid chills.

This caution must be well impressed on the patient, as those who are used to attacks of malaria are so apt to regard it as a trivial matter, and will not take proper care of themselves.

In an hour or two he will probably perspire freely, and should then be well rubbed down with hot towels and a clean warm suit put on.

Though for a day or two the case may be regarded as simple malaria, the nurse should take every opportunity to give the patient as much fluid nourishment as possible. This is rather a difficult matter, as, when the actual vomiting has stopped, he still feels sick and disinclined for food. The diet will be light, and it is most important that plenty of liquid should be taken.

When the acute symptoms set in the nurse must do her best to reassure the patient, for he at once realises the danger

THE NURSES' CLINIC.—*Continued.*

he is in, and if of an excitable or nervous disposition, the result can be imagined.

The frequent vomiting is very exhausting, but the patient's strength must be maintained as much as possible. Milk and barley-water or milk and hot water in small quantities every half-hour, or, if necessary, in 1-drachm doses every fifteen minutes, must be tried; or hot water or barley-water alone. If nothing can be kept down, the doctor will probably order rectal injections of saline solution to be given slowly with tube and funnel, and will give infusion into the tissues of saline solution.

Diarrhœa may be present, or the bowels may be very constipated. Large doses of calomel are generally given for the latter, followed by Carlsbad salts, and if necessary by enemata.

The temperature should be taken every two hours if it is above 104°, but in some cases it remains comparatively low. Death may occur from hyperpyrexia; the nurse must ascertain the doctor's wishes about sponging when the temperature rises.

The urine should be measured, and a specimen put up for the doctor every day. While the fever is high it will be scanty in quantity and nearly black in colour, but as the patient improves and the temperature falls, the urine increases in quantity and gradually returns to the normal colour.

In fatal cases the urine is passed in smaller and smaller quantities, becoming thicker until suppression ensues, and the patient dies of coma.

The fever leaves the patient extremely weak and exhausted, and his strength returns very slowly. He is also very anæmic, and must be careful to avoid sudden exertion, as sitting up quickly, etc.

The diet must consist entirely of milk and barley-water or milk only until the doctor orders more substantial food. The more fluid the patient takes the better.

When he is able to get about again he must be cautioned to wear warm flannel clothes, to avoid over exertion, and particularly to beware of chills.

It should be remembered that severe cases of this fever are nearly always fatal, so that attacks of malaria occurring in those who have been subject to blackwater before, or who have lived in the infected districts, should be watched most carefully, and every precaution must be taken during convalescence to prevent a relapse.

### Incidents in a Nurse's Life.

### "SUNNY JIM."

He did not look more than fifteen, but they told me he was a trumpeter in the Hussars as they carried him into the ward, where already the lights had been lowered for the night. Very fair and fragile he looked, lying there with closed eyes. And unconsciously I uttered my thoughts aloud, adding that he was surely too young for the rough life of a barrack-room. One of the two Hussars who had brought him turned half-indignantly at my words.

"He's made of the stuff they make heroes of, sister," he answered. "Every inch a soldier is Sunny Jim; we call him that 'cos of his ready smile."

"It's hard luck on him, sister," said the other man, shaking his head. "He'll be fed up when he finds us gone. Our regiment is going to India next week, and he's been fair mad to go. There ain't no homesickness about Jim; he's all grit right through. Even when the Captain's 'oss cotched him in his stomach he didn't holler; but the Captain took it orful bad, he did. He were fond of Jim; but the whole regiment is, for that matter."

The boy, half unconscious, owing to the pain of being moved, turned on his side and swore aloud.

The men exchanged an uneasy glance, and one stooped and shook the boy half-tenderly.

"Say, youngster, you're in orspital now," he whispered. "Don't you be so loose and free in your talk; there's a sister here."

The other man moved towards the door, but as he went he threw out an appeal on the boy's behalf.

"He ain't got no mother, sister, and it's mighty hard on him being left behind. Perhaps he needn't know the day we go; he'd take it to heart, poor Jim; he's been so keen on seeing India"

There was a tear in the man's eye, and his rough voice trembled as he spoke. The familiar sound brought a smile to Jim's face—the smile that had earned for him his nickname.

"One of you chaps pit my kit up," he called. "I'll be ready when my marching orders come. I say, let me sleep now. Oh, dear, how thirsty I am!"

In the days that followed he proved the truth of their words. Pain he had such as few have to suffer—pain that drove the colour from his lips and left him pinched and thin—but he met it unflinchingly, asking each day if he'd be ready to go with the regiment.

"The Captain, he said I was to be ready when my marching orders came," he explained. 'Bite on the bullet when it hurts,' he said. And I do; don't I, sister?"

Each morning when the bugles woke the men he would turn restlessly, asking suspiciously what day it was. Once he apologised for his eagerness to be gone. "I should lie quite content if a bullet had drilled me," he said sadly; "but I reckon it's tame to be kicked by a 'oss. There ain't no glory in bearing up, is there?"

I whispered there were heroes who never saw a battle-field, but he waved my words aside.

"I guess I'd like to see one," he cried, and drew in his breath with a whistle as the old pain began.

How I grew to dread that fatal day that was to send his regiment to India! How could I keep the knowledge from him and prevent him from hearing the tramp of the cavalry preparing for the march? But a wiser and kinder Hand than mine had planned to spare him, for the evening before Jim got his summons.

That night, after the "Last Post" had sounded, he sat up in bed cheering his comrades on to battle, jeering at their lack of enthusiasm, calling them slack and stronger terms heard in a barrack-room. He was in India then, feeling the heat and dratted flies, running for water, helping to carry the wounded out of the line of fire. Hard he worked, Sunny Jim, putting some of his pluck into others, but as the grey dawn of a winter's morning crept into the wards he was quiet. Then, dear lad, as the bugles called up the men who had got their marching orders, Jim got his—but not for India.

### Wants and Workers.

A nurse would be very grateful for a child's spinal carriage. The case is a very deserving one, and a small sum would gladly be paid. Address, Nurse Woodward, Stowe Hill Cottage, Lichfield.

### Central Midwives Board.

A meeting of the Central Midwives Board was held on Thursday of last week at Caxton House. Dr. Champneys presided, and there were also present Mr. Parker Young, Mr. Fordham, Dr. Dakin, Mrs. Latter, Miss Wilson, and Miss Paget.

October Examinations.

The secretary's report on the October examinations showed that out of a total of 446 entries, 346 midwives passed and 100 failed, the percentage of total failures being 22.4. Dividing the candidates into those entered from a training school and those prepared by private tuition the following result was obtained :—

| | Total. | Passed. | Failed. | Per cent. of failure. |
|---|---|---|---|---|
| Training Schools ... ... | 334 | 262 | 72 | 21.5 |
| Private Tuition ... ... | 112 | 84 | 28 | 25 |

The percentage of failures among the London candidates was considerably less :—

| | Total. | Passed. | Failed. | Per cent. of failure. |
|---|---|---|---|---|
| London Training Schools ... | 159 | 131 | 28 | 17.6 |

Wanted—A Welsh Translation.

Several communications were dealt with in the Standing Committee's report, and their recommendations, with a few modifications, were adopted. A letter from the Denbighshire County Council forwarded a request of the local supervising authority that a Welsh translation of rules and forms should be issued. It was decided to reply that the Board had no objection to the County Council "making its own translation for the information of midwives who are unable to understand English."

The case of Mrs. C. S. Selwood, of 68 Kew Road, Richmond, on whose behalf a memorial of twelve medical practitioners requested the grant of a certificate under Section 2 of the Midwives Act, has already come before the Board, who now adopted the recommendation of the Committee that Mrs. Selwood should be admitted to the examination, subject to the production of evidence that she had complied with the London Obstetrical Society's conditions of training.

Suggestions from the Provinces.

The Standing Committee presented recommendations with regard to suggestions arising from various sources. The Somerset County Council sought for observations upon certain proposals made to the local supervising authority respecting the supply of midwives in county districts. It was decided to inform the Council that the scheme in question was too fragmentary to warrant an expression of opinion, but that if submitted on completion it would receive consideration.

It was agreed to inform Dr. Middleton Martin, County Medical Officer for Gloucestershire, that the Board is unable to dispense with the keeping of the register of cases as prescribed by Rule 19a. Dr. Martin had pointed out that the Queen's Nurses were obliged to keep two registers—that required by their own institution and that of the C.M.B.—and he considered the former more business-like.

The secretary was instructed to inform Dr. Armstrong, medical officer of health, Newcastle-on-Tyne, that the Board, while approving his suggestions for instructing and supervising uncertified women practising as midwives and for securing the notification of births by them, had no jurisdiction to deal with this class of women.

"Suspension."

In reply to a letter from the Marchioness of Winchester, Vice-President of the Hants County Nursing Association, criticising the use of the word "suspend" and "suspension," it was decided to point out that these terms had definite technical meanings laid down by the Midwives Act and the rules, and to intimate that the Board could not deal with the matter. The Chairman remarked that there was, of course, no ground for supposing that the term objected to had any derogatory meaning.

### Appointments.

Brighouse Isolation Hospital.—Miss A. M. Whitehouse has been appointed nurse-matron. She was trained at King's Norton Infirmary, and has since been ward sister at St. Luke's Hospital, Halifax, and night superintendent at Mill Road Infirmary, Liverpool.

Brighton Throat and Ear Hospital.—Miss Mabel Ogden has been appointed matron. She was trained at Leicester Infirmary, where she was afterwards sister. She has since been in charge of the Vale of Clwyd Sanatorium, temporary matron of Bourton Cottage Hospital, sister of female surgical ward at University College Hospital, and housekeeping sister at Leicester Infirmary.

Camberwell Infirmary, London.—Miss Ellen E. Blackmore has been appointed staff nurse. She was trained at Fulham Infirmary, and previous to her training she was for two years at the Exeter Fever Hospital.

Cottage Hospital, Redhill.—Miss E. M. Olding has been appointed staff nurse. She was trained at the Seamen's Hospital, Royal Albert Docks, where she has since been staff nurse. She has also been attached to the East Ham branch of Plaistow District Nurses' Home. She holds the C.M.B. certificate.

Hackney Union Infirmary, Homerton.—Miss Emma Eliza Fraser has been appointed charge nurse. She was trained at Hackney Union Infirmary, where she has since been staff nurse.

Hayes Cottage Hospital.—Miss Ellen Hailey has been appointed matron. She was trained at Mile End Infirmary and the Uxbridge Cottage Hospital, and has since been ward sister, night sister, and home sister at Shoreditch Infirmary and temporary matron of Uxbridge Cottage Hospital.

High Wycombe Hospital.—Miss Grace M. Lloyd has been appointed matron. Since training, she has been sister at Lambeth Poor-law Infirmary, sister at Shoreditch Poor-law Infirmary, superintendent nurse at Braintree Infirmary, and sister at the North Eastern Fever Hospital, London. She holds the C.M.B. certificate. There were 89 applicants.

Isle of Wight Workhouse Infirmary, Newport.—Miss Isabella Lyon has been appointed superintendent nurse. She was trained at Birmingham Poor Law Infirmary.

Liverpool Convalescent Institution.—Miss A. Mulligan has been appointed lady superintendent. She was trained at Sir Patrick Dun's Hospital, Dublin, and has since been matron of the Borough Hospital, Wolverhampton, and matron of the Huddersfield Sanatorium.

Newcastle-under-Lyme Union Infirmary.—Miss A. Woodward has been appointed superintendent nurse. She was trained at the Bradford Poor-law Infirmary, and has since been ward sister and night superintendent. She holds the C.M.B. certificate.

Orthopædic Hospital, Redland, Bristol.—Miss Amy Robinson has been appointed sister. She was trained at St. George's Hospital, London, and for fever at Brighouse. She has since done two years' district nursing at Middlesbrough and has been staff nurse at the Corbett Hospital, Stanbridge.

### Everybody's Opinion.

We regret that in consequence of the pressure on our space we are compelled to hold over a number of interesting letters, including one from "A Richmond Guardian" on "Irrelevant Questions."

### "Cruel and Degrading Christmas Presents."

MATRONS' VIEWS.

Clara A. Ford, Lady Superintendent, Fever Hospital, Chertsey, writes : I was very pleased to see the letter by "Poor Penniless Pro," for of late years, owing to the almost compulsory giving of presents in hospital by nurses of all ranks, Christmas time as it approaches is looked upon with feelings of dread and horror. Therefore some readers may feel interested in hearing of what I, at last, have found the moral courage to do. It has been for some years the custom of the staff to present me with a gift and to give each other an exchange of presents. The majority of my nurses are unpaid probationers, who have no money to spare for any but their own nearest and dearest. This year I put up a letter in the dining-room, saying I should neither give nor receive presents from anyone in the hospital, explaining my reasons for so doing, namely, that the staff had other and more legitimate uses for their money, and I had the same reasons on my side. My letter was very nicely and, I feel sure, most thankfully received, and I and my staff are possibly better friends than we were a year ago. If only more matrons and heads of hospitals would do likewise, I think Christmas time would not be looked upon with the feeling that it is now by hard-worked and, in many instances, underpaid nurses.

"Sister Ellen" writes : I should like to say a few words regarding this vexed question of Christmas presents. We hear a good deal as Christmas approaches of "peace and goodwill," but I begin to think it non-existent in hospitals where the writers of some of the letters live. I quite agree that it becomes a tax if carried too far; no nurse ought to tip either servants or porters as they are not under their control, but I do believe it is a real pleasure for sisters and nurses to give their sixpences or shillings to purchase a gift for matron expressive of many favours received. Who is it but the matron who secures tickets for theatres, concerts, garden parties, picnics, and other pleasures, also helps the nurses to good posts when their training is completed, beside nursing them in sickness? My own staff have frequently said part of Christmas was the buying of some surprise for matron, and if stopped half of their joy would disappear. If any nurse is in need and cannot afford her mite she should be courageous and say so, when she would be admired rather than thought mean. I think expensive gifts should be discouraged, but I also think that where that bond of affection exists between matron and nurses it should be encouraged rather than checked. I may add that nurses whom I trained nearly twenty years ago write at Christmas and enclose perhaps their photo or some little gift, so that there are nurses who do it from love and not compulsion.

"The Superintendent of a District Nurses' Home" writes : The question I asked myself after reading "A Poor Penniless Pro.'s" protest against the practice of giving presents to those in authority was this : How is it that matrons and superintendents of nurses' homes have not long ago stamped out such an evil? Why have they not told their nurses long ere this that no presents of any description would be accepted by them? Had they done this, this grave scandal would not be with us now. We are constantly urged to advise nurses to make some attempt at providing for their old age. Of what avail are our words when our actions display such a lack of the sense of the fitness of things, for in receiving the presents from them we are encouraging a needless expenditure? Matrons want from their nurses something far more valuable than their money : they want their loyalty and their *esprit de corps*, which makes for the true happiness of the institute in which each respective nurse works.

"An Assistant-Superintendent" writes : May I be allowed to say a few words on the subject of official Christmas presents, speaking from experience in the past of hospitals where they are given and present experience of one where the custom has never been begun. Where official presents have been the rule it seems impossible that the first proposal to alter the custom should come from the nurses. It should be the matron's part to tell her nurses as a body that she does not consider the custom a good one, and that it is her wish that official presents should cease to be given. If this were said decidedly and kindly, and it were explained to the nurses that the matron much preferred receiving their good wishes only, there would be no awkwardness or ill-feeling. The question would thus be settled in the simplest way.

SISTERS' VIEWS.

"X. Y. Z." writes : While sympathising heartily with "Poor Penniless Pro." in her complaint of the hardship which compulsory present-giving inflicts on the givers, I would remind her that it is not the "pro." only, as she seems to think, who suffers under this tax, nor are the presents given only to "those in authority." She will find, probably, that the ward sisters, in addition to contributing to the funds for the matron's present and the porters' and servants' Christmas-boxes, makes a gift to each nurse in their wards, as well as to their ward maids. They also bear the chief expense of the decoration of their wards, buying plants and flowers, and hiring fairy-lamps, Chinese lanterns, and other such things. I have been a ward sister, and so I know. The matron often gives each individual nurse a small present, and sometimes a handsome Christmas cake for their tea-table. Hospitals, of course, differ in the amount and extent of Christmas present-giving. At one of the highest standing in England the matron never allows anything but a box of chocolates to be given her, and the contributions to this present must not exceed the modest sum of threepence. Money-boxes are placed on the nurses' dining-tables "For the maids' and porters' Christmas-boxes," and the pennies dropped in are truly "voluntary contributions." No other wholesale present-giving takes place, except that every man, woman and child in the hospital, doctor, nurse, patient and servant, has something off the Christmas-tree, and the Christmas Fund is collected outside the hospital, amongst the subscribers. On the other hand, there are hospitals where collections seem to be perpetual, for gifts to doctors and sisters on leaving, annual presents to the matron, on her birthday, besides Christmas presents, and so forth. And, as "Poor Penniless Pro." says, it is considered mean not to give, and giving may mean getting into debt. If you can put a stop to these abuses you will, indeed, deserve the gratitude of many a hard-up nurse. Surely the committee, or board of management of each hospital, should deal with the question. It would be very simple to "strictly forbid any collection of money for the purpose of present-giving at Christmas or any other time, by any member or members of the hospital staff, nursing or domestic, without the express sanction of the committee or board," and to add that any application for such permission must be in writing and be presented by the matron or senior medical officer.

"Sister B." writes : It was with great indignation I read "Poor Penniless Pro.'s" letter in this week's Hospital. If she finds she cannot afford to join in the various subscriptions which take place in most hospitals this time of the year, why not say so? I am sure it would be much nicer than airing her grievance before the public. Any hospital I have been in, either as "pro." or sister, we never collected for doctors' Christmas presents, and whoever we gave to we did it freely and with great pleasure. I am sure no matron or sister would like to think they received presents from their staff under such circumstances as "Poor Penniless Pro." describes. Christmas would not be Christmas if we did not give each other some little remembrance to mark the day from other days. I myself would not like to have the old custom changed, though I have nothing but what I earn. I must say that my greatest pleasure is buying my little Christmas gifts for my "pros." and fellow-workers, but I never allow my "home" people to suffer, or run myself into debt. I think it is very foolish for anyone to give beyond their means.

"Charge Nurse" writes : "Poor Penniless Pro." has my full sympathy, and I can fully endorse her facts from my own experience. I was trained for three years in a large provincial infirmary, where the practice of giving to those in authority was a severe tax on the pockets of the nurses. Each year we gave to the matron, assistant matron, night sister, home sister, ward sister, home maids, dining-room maids (the latter being quite distinct from the home maids), porters, and postmen. When these lists are passed round the nurses do not care to be thought mean, therefore those who are near and dear to us have to be put off with very little—sometimes nothing. I may add that the "pros." in their first year received no salary. I sincerely hope this question will be thoroughly considered in the interests of the "poor penniless pros."

"Would-be Sister" writes: It is not by any means only the "pros." who suffer from the practice of giving Christmas presents to those in authority. As ward sister in a hospital, I think the demands on one's purse are even greater in proportion to one's salary. And in some cases you will be almost obliged to contribute to presents for people for whom you have little respect, let alone regard. Of course, it is much more done in some hospitals than others, and the whole thing rests with the matrons. I was once in an institute where the matron refused to accept even the smallest present from the nursing staff. It makes it almost impossible for a nurse without a small private income to stay as sister in a hospital where it is the usual thing to contribute to something for matron (you are told what the sisters usually subscribe), to give a small gift to each sister and to each nurse working in your ward. Then there is your wardmaid, besides porters, maids, etc. A sister's expenses do not end here. If she wants her ward to look pretty she invariably spends a certain amount on decorations, and she also gives "teas" two or three times in the Christmas week, and she loves doing it if she has the wherewithal; but it cannot be done on £30 a year if friends and relations are to be remembered at all, because I have tried it.

NURSES' VIEWS.

"One who Likes Fair Play" writes: Having taken The Hospital now for some years, it is with regret and indignation that I read "Penniless Pro.'s" letter of to-day. I cannot help but write you and sincerely hope that you will grant a small space for my "opinion." First, "Penniless Pro." cannot realise the harm and embarrassment she may cause to many matrons, sisters, and nurses. During my three years' training I can conscientiously say I was never asked to contribute towards presents for matron, medical officers, maids, or porters. Christmas is always an expensive time in hospital, but the expense is voluntary, and I think I may state that not only voluntarily, but with greatest pleasure both ward sisters and nurses give and receive presents from each other, and also spare no expense and labour in making Christmas Day a red-letter day in the lives of everyone in hospital. I think that "Penniless Pro." has had very little experience in these matters, or else she is one of those people who go through life with a grievance. These, I am thankful to say, are few and far between in the nursing world. The choosing of sister's present was the greatest of our few pleasures in the wards, and was looked forward to for weeks. I am sure that there are numberless nurses who would endorse my opinion.

### Death of Sister Eliza.

On Saturday, December 1, there passed away at St. Saviour's Hospital, Osnaburgh Street, one who had given a long life to the service of God and man. Miss Crofts—known to the world as Sister Eliza—joined the All Saints' Sisterhood in 1856, and was the fourth to unite herself to that community, which has now such a long roll of membership. At the age of eighteen she had devoted herself to work among the poor in Brighton under the late Rev. W. Gresley. Although never receiving what is now considered full training as a nurse, her extraordinary natural ability and great love for this work enabled her to render substantial aid to the profession and to win the high esteem of many leading medical men. Her training she gained by experience. Sister Eliza helped at the London Hospital during the time of the cholera outbreak in 1859. In 1870, together with the Mother Foundress and six other sisters, she nursed the sick and wounded in the Franco-Prussian War from February to September, her patients being of various nationalities. The sisters were attached to Dr. Franks' ambulance, who placed a high value on Sister Eliza's services, and always singled her out to assist him in his operations. She and the Mother Foundress received the Iron Cross in acknowledgment of their services. Whilst living at the All Saints' Home, Sister Eliza tended the incurables, night duty being her particular share of the work. She organised and opened a home specially intended for penitent women, known as the St. Agnes' Hospital, which was at first situated in Newman Street, and afterwards in Margaret Street. This hospital she superintended, helped by other sisters, for seventeen years. She nursed the Mother Foundress during her last illness, and it was then that she came into contact with Sir William Jenner, who had a very high opinion of her ability, and strongly recommended her to attach herself to St. Saviour's Hospital. This she did in 1892, and laboured unremittingly until she had raised it to its present position. She was always ready and anxious to move with the times, and gladly adopted the recommendations of the medical and surgical staff as to improvements and additions. In the many letters of condolence received from medical men, they speak of her, not only as a great friend, but also in terms of profound respect.

### Leeds and the Pension Fund.

There was a large gathering of nurses at the Philosophical Hall, Leeds, on Friday last, when Mr. Charles Wilson, J.P., member of the Leeds City Council, presided at a meeting held to hear an address from the Secretary of the Royal National Pension Fund. He was supported, amongst others, by Mr. Richard Wilson, Treasurer of the Leeds Trained Nurses' Fund; Mr. Edward Lawson, the late Chairman of the Leeds Sanitary Committee; Dr. Heald, its present Chairman; Dr. Pearson, Medical Officer of the Leeds Fever Hospital; Councillor Thomas Woods, Chairman of the Harrogate Wells and Baths Committee.

In his opening remarks, Mr. Dick said that while naturally he would deal chiefly with the Society which he represented, he was in reality an agent—incidentally, an agent not working on commission—of any and every financially sound, properly managed thrift agency through which nurses could make provision for their future.

Mr. Thomas Woods (Inspector for Yorkshire of the Prudential Assurance Company), in proposing a vote of thanks to the Chairman, said that, although he was concerned in running an opposition show, as it were, he was pleased to testify to the pleasure he had in supporting the Pension Fund. As one interested in life assurance and earning his living from the profits of life assurance, he ought to know something about the question. The manager of the largest British Life Office had drawn his attention to the Fund and had asked him to explain what he thought about it. What he had to say was, that it was impossible for the ordinary Life Assurance Office to compete with the Pension Fund. The gentleman to whom he referred was himself on the Board, which is an honorary one, and all the other members were connected with the biggest Insurance Offices, the biggest banks or the biggest commercial houses. The Fund was, therefore, worked by the best men in the country, and the cost of administration on an average did not exceed four per cent., which is extremely low. He referred to the investments as being all gilt-edged and the Fund as absolutely safe and above suspicion.

Mr. Edward Lawson seconded the motion.

Dr. Heald, in supporting it, expressed the hope that nurses would take the opportunity of making provision for their future.

In answering a question which had been put to him by Mr. Lawson, Mr. Dick stated that he was glad of the opportunity of saying that for years past at each annual valuation the securities of the Fund had shown an appreciation.

After the meeting the nurses adjourned to tea, the matrons of the hospitals and infirmaries in Leeds acting as hostesses.

### Christmas Books.

Unlimited leisure rarely falls to the lot of a nurse, and if this be true at ordinary times it is doubly so in the Christmas season. Therefore a few details of some of the newest publications and their prices may be of practical help to those who wish to make purchases, but having no time to go out and choose, must perforce order by post.

### Nelson and Son.

"Uncle Remus" (5s.) A very handsome gift-book, which deals with the adventures of Brer Rabbit, Mr. Fox, and other animals, has twelve beautifully coloured pictures and upwards of eighty humorous pen-and-ink illustrations. Children who are lovers of animals will also enjoy the amusing letterpress. "Doris Hamlyn" (2s.), by R. O. Chester, is a love tale of an exciting character, where the heroine is able to save a prisoner from the gallows, and is herself saved from a burning theatre. From the pen of R. S. Warren-Bell there is a story described as "for boys and old boys," entitled "The Duffer" (5s.), The hero begins by being expelled from school, but shows later that he has plenty of "grit." His sister Molly is a winsome English lass, and one hardly wonders at the artist's admiration for her. Boys of a mechanical turn of mind will revel in "How It Works," by Archibald Williams (3s. 6d.). It is a handsome volume, lavishly illustrated, and it explains in simple language how, for instance, the mechanism of the keyless watch, the free wheel of the bicycle, or the cream separator each of them works. Wireless telegraphy, the steam turbine, a piano, a phonograph are all introduced, and the handy reference index will be found exceedingly useful.

### Wells Gardner, Darton and Co.

No prettier book for children has ever been written than Evelyn Everett Green's "Dickie and Dorrie" (2s.), and we distinctly envy those who still have the pleasure of perusing it for the first time. The lesson it teaches will probably impress itself deeply, though unconsciously, upon its young readers. The new edition of "The White Stone" (3s. 6d.), by Herbert Macilwaine, is certain to be in demand. It is a fascinating narrative of life in the Australian Bush, varied by the hero's school experiences in England, and boys will be sure to enjoy it. Another good book for boys is by William A. Bryce and H. de Vere Stacpoole. It bears the title of "The Golden Astrolabe" (3s. 6d.), is well illustrated by A. S. Boyd, and abounds with hairbreadth escapes and marvellous adventures, the last chapter leaving the heroes "healthy, wealthy, and wise." Those who prefer home rather than foreign adventures will like "The Boy Tramp" (2s. 6d.), by Thomas Cobb. All girls love Raymond Jacberns's books, and they will be delighted to welcome a new one from the same pen, "Ruler-in-Chief" (1s. 6d.), which relates the attempts of a fifteen-year-old elder sister to act as deputy mother to a family of four, who are sent away from home under her charge. There is a subtle charm about "The Fiddle String" (2s. 6d.), by R. H. Bretherton, and the story how little Phyllis helps to introduce a musical genius to the world runs on distinctly original lines. Those who want to give a younger child a present could not do better than purchase "Why-Why and Tom-Cat" (3s. 6d.). "Why-Why" is the name bestowed upon a little girl by the Tom cat when she goes with him in a dream to his own land, because she asks so many questions. The authoress, "Brown Linnet," writes for children as if she loved them, and Gordon Browne's illustrations are delightful. A wee volume for those about to be confirmed is "Chrissie's Confirmation" (6d.), by Evelyn Hunt, and "The Little Major," by M. S. Haycraft, at the same price, is admirable.

"The Railway Children," by E. Nesbit (illustrated, 6s.), is a really delightful child's book. The illustrations are charming and the adventures of Roberta, Peter, and Phyllis are of an out-of-the-way kind, but as they are quite natural children, everything that happens is just what one might expect under the circumstances. They have also a father and mother of the same sensible sort as themselves. "Mother," we read, "did not spend all her time paying calls on dull ladies, and sitting dully at home waiting for dull ladies to pay calls on her. She was almost always there ready to play with the children and read to them and help them to do their home-lessons. They had also a father who was never cross, never unjust, and always ready for a game. In addition, these lucky children had everything they needed—a lovely nursery, a kind and merry nursemaid, a Mother Goose wall paper, and a dog called James, who was their very, very own."

### Christmas Numbers.

The "Sunday At Home" commences with a short story by Ian Maclaren, entitled "A Soldier of the Lord," and, as well as two other complete stories, gives an interesting account of the culture of bananas in Uganda, and a poem by the Bishop of Durham. It is a capital sixpennyworth. There is a warm welcome assured for the Christmas Number of the "Boy's Own Paper," which is stuffed full of seasonable fare for its many readers. Moreover, a clever boy who carefully reads some of the pages will find that he, in his turn, can provide amusement for his elders in several directions. The tricks are well described, and so amply illustrated that they are quite easy to follow. The "Girl's Own Paper" in its turn provides plenty of interesting reading, notably "The Making of Elsa" and "Martin Croft's Daughter." The hints on Tableaux Vivants and some novel methods of giving Christmas presents should prove exceedingly useful just now.

### Christmas Presents.

### MAPLE'S CHRISTMAS PRESENTS.

Those who can only afford an hour or two to buy their Christmas gifts cannot do better than pay a visit to Messrs. Maple and Company, Tottenham Court Road. Here under one roof a varied collection of presents can be seen, ranging in price from a few pence to some hundreds of pounds, and in size from a thimble to a suite of furniture. I was specially attracted by the real silver buckles and waist-clasps in beautiful new designs, some as low as 11s. 6d. in price; and also by some charming lace brooches. These are put into neat little cases, and are four in number, two large and two smaller. The set of "Fly" brooches, composed of pearls and sapphires, are two and a-half guineas, those with turquoises about a sovereign less. They would make a charming gift. The crocodile leather silver-cornered blotting-pads for a writing table are suitable to give either to a lady or a gentleman, and start from 10s. The quaint fire-screens in antique copper and wrought iron are quite works of art; whilst the pretty ladies' work-tables, in satinwood and mahogany, with silk bags to hold the needlework and drawer fitted with divisions, are a revival of an old custom, both useful and ornamental. It is impossible to mention a tithe of all the dainty things offered for sale in these handsome showrooms, but my advice is, "Go and see for yourselves."

### A Book and its Story.

### MRS. F. REYNOLDS' NEW NOVEL.*

"Hazel of Hazeldean" is a gracefully written novel, and the character of Hazel a charming delineation of a girl, who, by the dying wish of her father, has been brought up as a boy, in order that she may inherit the title and estate of Hazeldean. Mrs. Reynolds has treated a difficult situation with much ingenuity, and if the possibility of it taxes the unimaginative reader's powers it will, on the other hand, delight those who like their fiction to have an old-fashioned plot with a mystery in the background to keep the attention alert until it is solved. Here the secret is well kept, and an artistic ending, cleverly managed, brings the story to a satisfactory conclusion.

The book begins with a last interview between Raimond Hughenden and the family lawyer, who has come abroad in search of the missing heir. Sir Hazel, the present owner of Hazeldean, is old, and anxious to leave his affairs in order before his death. Many false leads had been followed by Farrer, the lawyer, in quest of the heir. At last correct information has enabled him to find Raimond Hughenden. It comes too late to be of use to Hughenden, but in time to secure the succession to his only child, Hazel. Farrer finds Hughenden mortally ill in charge of an old Frenchwoman who has been to his child nurse, mother, and playfellow. It is with difficulty that Farrer gains admittance to the sick man. The Frenchwoman only allows it after a pencilled note has been written and sent in to him. Despite the dust and heat and the oddness of his reception, he is not without a sense of satisfaction that a long and weary quest had been brought to a successful conclusion. "To find him alive," he thought—"above all to find he has a son" with a glance at the tiny figure which is busy following with eager eyes the rapid disappearance of a bright green lizard into a crevice in the sunbaked earth. "A fine little chap, too," the lawyer continued, fixing his gold-rimmed eye-glasses into position. "This will be good news for Sir Hazel, if," with a sigh, "he lives long enough for it to reach him." His meditations are interrupted by the noiseless return of the Frenchwoman, who announces that her master is waiting to see him. Then he follows her through an arched doorway hung with mosquito-netting, and as she pauses and turns for a moment towards him, he is struck by the pitiful workings of the broad face visible even in the dim light of the interior. Having ushered her visitor into the presence of the sick man she retires. "Farrar," said a weak voice, "it is like a breath of dear old English air just to hear your name. Come here and let me see an English face, a good old English face once more." The voice comes from behind a tall screen standing between the window and door, which in the now ending daylight added to the duskiness of the room. "Stretched on a low bamboo couch, haloed by the red gleam of the departing sun, lay the long, gaunt figure of a man. . . . Only the eyes, playing in their deep sockets, still shone virile, glowing yet with the concentrated power of life, as though the soul—fast being ousted from a failing body—had made in them its last gallant despairing stand. By those eyes, and those alone, after half a lifetime of separation, the lawyer would have recognised a scion of the family whom from boyhood he had been taught to revere and serve. . . . how wasted and wrecked they proclaimed him a Hughenden still." He lingers long enough to dictate his wishes as to the guardianship of the child, what is to be shared by Farrar and Adèle Deschamps, the Frenchwoman. Of the child's mother nothing is divulged, except what is found in the certificate of her marriage to Hughenden at a Mission church in one of the Pacific Isles. The end comes quickly after the father has had a document drawn up to secure the safety of his child's future. "At last all duly signed and sealed," with fingers that trembled, as they touched the motto of the house, "Ad Finem," Raimond Hughenden sank back upon his pillows, white and exhausted. Before his death the Frenchwoman had promised Hughenden that she would never leave the little Hazel, and that a tutor should be engaged when the child was old enough. Instead of the usual public-school education, it was to be brought up under the tutor's direction. When all is over the child and her nurse-guardian go at once to England, accompanied by the family lawyer. A graphic description is given of the mansion and surroundings of Hazeldean. "Hazeldean Abbey had been in the Hughenden family since the sequestration of the Monastries by bluff King Hal. The house itself was wide-spreading, built of splitflint and faced with ruddy brick; guarded by massed beech-trees from east and north, its noble front faced the west, and at sunset its many windows blazed ruddily with a cosy suggestion of family life within. To the west and south lay smooth lawns broken by rose-arches and monstrous ivied baskets filled with bright-hued blossoming plants. The carved inscription, 'I marke onlye sunnie houres,' on the ancient sundial was almost hidden by clustering Penzance briars, with sweet, old-world fragrance and heart-shaped petals. . . . Beyond the lake stretched the abbey meadows, shaded here and there with groups of lime and sycamore, about whose feet bloomed in the springtime the earliest celandines." Visitors approaching the mansion from the high road entered by a wide stone gate-way, with massive weather-indented balls surrounding the pillars on either hand, and richly wrought-iron gates. It was by this entrance that the tutor, Mark Taunton, engaged to superintend the studies of the little Sir Hazel, first sees Hazeldean. He meets the child wandering in the grounds. "Are you Sir Hazel Hughenden?" asked Taunton. For the life of him he could not help his voice falling rather flat; for the place seemed to him in many ways so desirable, but the boy so very small. "Yes, I'm Sir Hazel," answered the mite, rather wistfully, the other thought. "What is your name?" . . . "Mine is Mark Taunton." "I like your name, and I like your face," said the little one, looking earnestly into the blue-grey eyes that were gazing so searchingly into his. So the two start with mutually well satisfied impressions of each other, which time and fuller acquaintance verifies. Mark Taunton is a distant connection of Hazel's. His entrance into the daily routine at Hazeldean marks an important date in the future of the little heir. Between him and Mark a great friendship springs up. From this point the novel gains in interest until the *dénouement* arrives. As a harmless, and from the point of plot, exciting book, we cordially recommend "Hazel of Hazeldean." It has many alluring descriptions of places, and the style dainty and fresh, has the charm natural to Mrs. Fred Reynolds' writing. She has written stronger books, but "Hazel of Hazeldean" compares favourably with them from another point of view, and the skilful handling of a difficult and delicate problem compensates for any minor deficiencies.

* "Hazel of Hazeldean." By Mrs. F. Reynolds. (Hurst and Blackett. 6s.)

### Notes and Queries.

**REGULATIONS.**

**The Editor is always willing to answer in this column, without any fee, all reasonable questions, as soon as possible.**

**But the following rules must be carefully observed.**

**1. Every communication must be accompanied by the name and address of the writer.**

**2. The question must always bear upon nursing, directly or indirectly.**

**If an answer is required by letter a fee of half-a-crown must be enclosed with the note containing the inquiry.**

*Maternity Nursing.*

(122) I wish to gain some maternity nursing training before I go to Canada. Are there any lectures in Birmingham on this subject?—*Montagu.*

Would it not be as well to gain your experience in Canada? You would then be more likely to secure a clientele. As to lectures in Birmingham, write to the Matron of the General Hospital in that city, and ask her advice.

*Snoring.*

(123) I am a maternity nurse. I snore in my sleep. Can I cure this?—*Eight.*

Consult a medical man, as there may be a removable cause connected with your throat or nose.

*Massage.*

(124) Can you recommend a book on face massage, illustrated?—*Carnation.*

We know of none solely for this purpose, but Mrs. Palmer's "Lessons on Massage," price 7s. 6d., may help you. It can be obtained from the Scientific Press, 28 Southampton Street, Strand, W.C.

*C.M.B.*

(125) Is the Royal Maternity Hospital, Edinburgh, recognised as a training school for the C.M.B., and is the training equal in quality to other institutions preparing for the C.M.B.? Can I get a post-graduate training in gynæcology, and is a special certificate issued for this branch of nursing?—*Steady.*

The institution is recognised by the C.M.B. We cannot make comparisons. In England post-graduate teaching is not generally to be secured, but you might be received into a hospital for women for a shorter time than that necessary in a general hospital. You could not get a certificate, though no doubt the matron would help you by giving you a reference.

*Massage.*

(126) Can you tell me if there is a school of massage either in Birmingham or Sheffield?—*Sister R.*

If you write to the Incorporated Society of Trained Masseuses, 12 Buckingham Street, Strand, W.C., they will probably be able to tell you.

*County Council School Nurse.*

(127) Will you kindly tell me the qualifications for a Board School nurse, and their duties?—*M. M. H.*

Write to the London County Council, Spring Gardens, S.W.

*Nursing Abroad.*

(128) Kindly tell me when an experienced nurse should best apply in order to get work for the winter in the Riviera or Rome.—*Swallow.*

Refer to "How to Become a Nurse" under "Nursing Homes Abroad," published by the Scientific Press, 28 Southampton Street, Strand, price 2s. 4d. post free.

*Women Sanitary Inspectors.*

(129) Can you tell me the necessary qualifications to obtain a post as lady sanitary inspector; also where to apply for information about examinations for such posts?—*A. M. W.*

Write to the Sanitary Institute, Margaret Street, W.

*Dispensing.*

(130) Can I learn dispensing by correspondence, or could I learn from the local chemist?—*Cornwall.*

We should not advise you to learn by correspondence, unless it is quite unavoidable. As to the chemist, it depends very much on his qualifications. Write to the Pharmaceutical Society, 17 Bloomsbury Square, W.C. Ask them to advise you. Many chemists are members of the Society.

**Handbooks for Nurses.**

| | Post Free. |
|---|---|
| "How to Become a Nurse: How and Where to Train." | 2s. 4d. |
| "Nursing: its Theory and Practice." (Lewis.) ... | 3s. 6d. |
| "Nurses' Pronouncing Dictionary of Medical Terms." | 2s. 6d. |
| "Complete Handbook of Midwifery." (Watson.) ... | 6s. 4d. |
| "Preparation for Operation in Private Houses." ... | 0s. 6d. |

Of all booksellers or of The Scientific Press, Limited, 28 & 29 Southampton Street, Strand, London, W.C.

### For Reading to the Sick.

### THE PILGRIM WAY.

The way is dark, My child! but leads to light;
I would not have thee always walk by sight;
My dealings, now, thou canst not understand:
I meant it so; but I will take thy hand,
And through the gloom,
Lead safely home
My child.

The path is rough, my Father! many a thorn
Has pierced me, and my weary feet are torn;
And bleeding marks the way. Yet Thy command
Bids me press forward. Father, take my hand;
Then, safe and blest,
Lead up to rest
Thy child.

The path is rough, My child! but O how sweet
Will be the rest for weary pilgrims meet!
When thou shalt reach the borders of that land
To which I lead you, as I take thy hand,
And safe, and blest,
With Me shall rest
My child.

*Anon.*

The faith which ever waits on God is ever gaining new gifts. In trial and care we feel faith's work real. We count up our needs, and weigh the load under which we almost sink: then we can in thoughtful trust lay hold on the Divine help, and be at rest. He who gives those great things by which souls live, will add the lesser things which we need day by day. He who bare our sins, bare also the burden of the griefs that try in this world of want and sorrow. That love that bends down to each, and makes the want of every heart its own, does not tire of caring for the weary. If we have found out our own helplessness to carry what is laid on us, and we feel that we totter and go feebly, this need not be: for Christ is ready to be more and more our righteousness, and to make us strong in the power of His might.

We can look up to the skies and trace in their glories the hand of our Father. He whose mind planned all this, moulds us by His Spirit for His holy ends. He who marked out the courses of the stars, gives us our law and rule; and is with us in power, training us that we may move evenly and surely and safely according to His will. God has visited us to make His abode with us, and to impart to us of His own glory. No one is overlooked or forgotten in the crowd. We were in His mind before He called us into being. Though we are thoughtless of Him, He is always mindful of us. He visits us; sometimes so quietly that we hardly mark His presence, sometimes so as to make it plain that the great God remembers us.

God works not chiefly for man's eye. From higher worlds the wonders of this world may delight beings who see in them more than our minds grasp. Angels, who carry out God's will in Nature, and who minister to the heirs of salvation, know more of God's far-reaching aims, and the meaning of all least things. There is joy over one sinner that repents and yields to God's forming hand. God rejoices as His purifying work goes on. That work in us is the bringing out in us the likeness of Christ. In Him we behold the glory of God; and from glory to glory more like His, we are changed.—*Anon.*

